# Transglutaminase-Catalyzed Bottom-Up Synthesis of Polymer Hydrogel

**DOI:** 10.3389/fbioe.2022.824747

**Published:** 2022-03-22

**Authors:** Enping Lai, Binyu Bao, Yifei Zhu, Haitao Lin

**Affiliations:** Guangxi Key Laboratory of Green Processing of Sugar Resources, College of Biological and Chemical Engineering, Guangxi University of Science and Technology, Liuzhou, China

**Keywords:** biocatalytic synthesis, transglutaminase, polymer hydrogel, bottom-up approaches, enzyme catalyzed reactions

## Abstract

Enzyme catalysis has attracted increasing attention for application in the synthesis of polymer hydrogel due to the eco-friendly process and the devisable catalytic reaction. Moreover, bottom-up approaches combining enzyme catalysts and molecular self-assembly have been explored for synthesizing hydrogel with complex architectures. An enzyme widely distributed in nature, transglutaminase (TGase) has been confirmed to catalyze the formation of isopeptide bonds between proteins, which can effectively improve the gelation of proteins. In this mini-review, TGase-catalyzed synthesis of polymer hydrogels, including fibrin hydrogels, polyethylene glycol hydrogels, soy protein hydrogels, collagen hydrogels, gelatin hydrogels and hyaluronan hydrogels, has been reviewed in detail. The catalytic process and gel formation mechanism by TGase have also been considered. Furthermore, future perspectives and challenges in the preparation of polymer hydrogels by TGase are also highlighted.

## Introduction

Enzymes are biological catalysts essential for life processes and catalyze many vital reactions selectively in nature and living cells (Behabtu and Kralj, 2020). For materials synthesis, natural polymer materials such as proteins and polysaccharides are synthesized utilizing enzymes in nature. Similarly, synthetic polymer materials including polyesters, polycarbonates, and polyphosphates have also been synthesized using enzyme-catalyzed processes (Varma et al., 2005). For biomedical application, natural and synthetic polymer materials with desired molecular architecture and biocompatibility are required to meet the specific needs (Yan et al., 2020). Since enzyme catalysis has proven to be an effective synthetic method for polymers with complex architectures and mimicking extracellular matrices, increasing interest in the enzyme-catalyzed synthesis strategy has been developed.

As a biomedical polymer material, hydrogels with a three-dimensional (3D) network structure can mimic cell and tissue culture environments, leading to application in tissue engineering, wound healing, cartilage repair and other fields (Sharma and Tiwari, 2020). Additionally, hydrogels can provide multidimensional ion or biologically active molecules transport pathways for the delivery and controlled release (Lai et al., 2019; Cui et al., 2021). Although hydrogels have been traditionally synthesized using chemical and physical methods, rapid developments in enzyme-catalyzed synthesis technology are emerging. Compared with physical and chemical crosslinking methods, enzyme-catalyzed reactions can react with target molecules directly in mild condition and mimic *in vivo* biosynthetic processes, making the active molecules safer during the hydrogel synthesis process (Li et al., 2020).

TGase have received increasing attention due to the ability to catalyse the coupling of a free lysine amine group from a protein or peptide bond to a deamidated glutamine protein or peptide-bound γ-carboxamide group (Guebitz and Nyanhongo, 2018). These are important catalysts for the formation of protein-based hydrogels with varying tunable properties, and remarkable achievements of hydrogels synthesized by TGase based on natural and synthetic molecules have been made. The present paper reviews the TGase-catalyzed reactions for the synthesis of polymer hydrogel with a bottom-up strategy. The catalytic crosslinking and gelation mechanism of TGase is discussed, and future perspectives and challenges in the preparation of polymer hydrogel by TGase-catalytic reaction are also provided.

## Transglutaminase-Catalytic Process and Gel Formation Mechanism

### Sources and Characteristics of Transglutaminase

Transglutaminase (TGase) is an acyltransferase, which catalyzes the amide-transferase reaction between the γ-amyl group of glutamine residue and the ε-amino group of lysine in protein, resulting in form the ε- (γ-glutamine) -lysine heteromorphic peptide bond (Fisher et al., 2017). It can also make polymers containing amino and glutamate functional groups undergo cross-linking reactions (Maddock et al., 2020). TGase is widely distributed in nature and can be isolated and extracted from animals, plants and microorganisms. Notably, the coagulation factor XIII in human blood is also a kind of TGase. With the enzyme, fibrin stabilizing molecules were formed by the cross-linking reaction between fibrin molecules, leading to blood coagulation (Schmitz et al., 2020). Therefore, TGase has a unique effect in catalyzing cross-linking of protein molecules. TGase from animal tissue has few sources, low yield, complex separation and purification process. More importantly, the TGase from animal tissue cannot show its catalytic activity without calcium ions (Savoca et al., 2018). Compared with the TGase from animal tissue, TGase derived from microorganisms (microbial transglutaminase, MTGase) can be directly secreted into the culture medium, which is easy to separate and purify with cheap raw materials and a short production cycle (Xue et al., 2020). For use, MTGases can show their activities without calcium ions and have a wide range of optimum temperatures and pH values, which have advantages over the TGase from animal issues.

### Transglutaminase-Induced Gel Formation Mechanism

Due to the excellent properties of TGase, it can be used to modify the protein. The catalytic cross-linking effect is beneficial to the gelation of protein molecules, leading to improving elasticity, adhesion, water retention and other qualities of the protein (Gharibzahedi et al., 2018). For protein molecules, the catalytic effect of TGase is mainly by three ways, including amine import, cross-linking and deamination. TGase catalyses the acyl transfer reaction between glutamine residues γ-carbonyl and primary amine in the peptide bond, introducing lysine into the protein (Fatima and Khare, 2018). When the lysine residue γ-amino is used as the acyl receptor, the covalent bond of ε- (γ-glutamyl) lysine is formed between protein molecules, which means the protein molecules are crosslinked. In addition, water can become the acyl receptor in the absence of primary amines, and the γ-amino group is deaminated into glutamate residues (Miwa, 2020).

**FIGURE 1:**
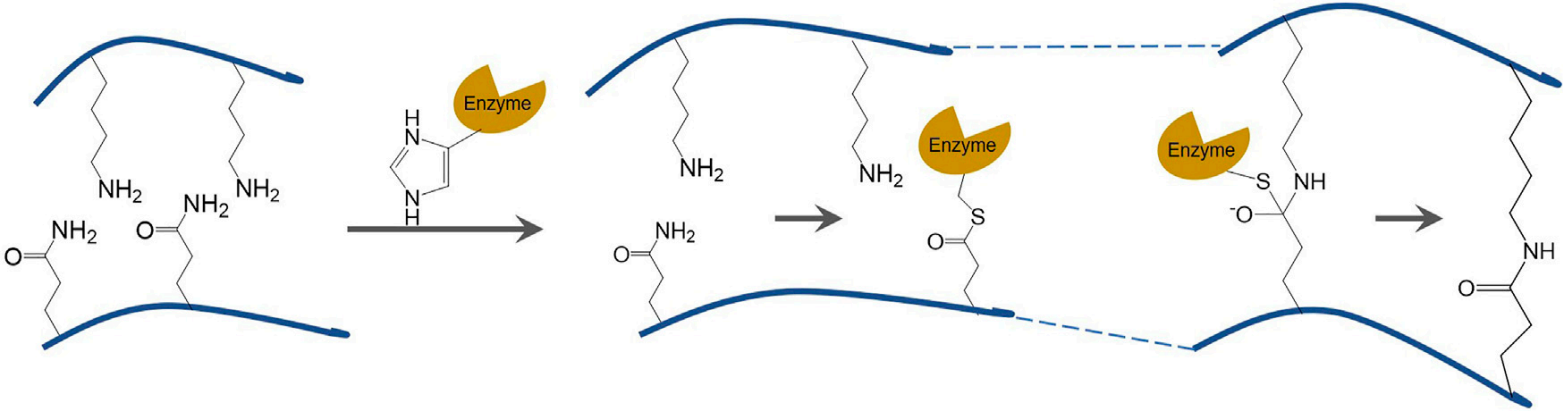
Schematic diagram of TGase catalyzed crosslinking reaction.

A gel is a substance with a specific spatial structure formed by the aggregation of denatured protein molecules. At the microscopic level, the gel is represented by the equilibrium of attraction and repulsion between protein molecules. It is represented by the formation of semi-solid inverted colloidal substances without flow macroscopically (Wang J. et al., 2021). In general, the covalent crosslinking reaction catalyzed by TGase occurs faster than the acyl transfer and deamidation reactions. Therefore, TGase can induce the formation of highly elastic and irreversible gel structures even at low protein concentrations (Duarte et al., 2020). Typically, TGase catalyzes the formation of ε-(γ-glutamyl) lysine covalent bond between the γ-hydroxylamine group of glutamine residues in soybean protein and the ε-amino group on lysine in wheat protein (Qin et al., 2017). Figure 1 depicts the schematic diagram of TGase catalyzed crosslinking reaction, and the formation of covalent bonds between a free amine group and the γ-carboxamide group. These covalent bonds are highly resistant to proteolytic degradation and more stable than the hydrogen bond and disulfide bond, resulting in the formation of stable polymeric networks.

## Transglutaminase Catalysed Formation of Polymer Hydrogel

**FIGURE 2:**
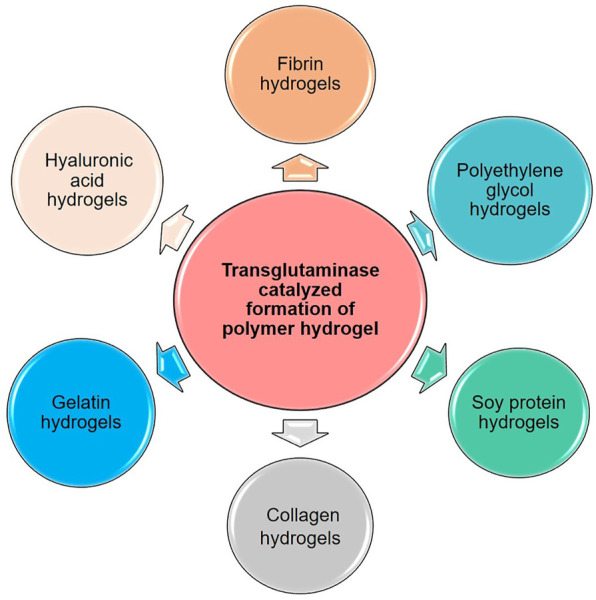
Various polymer hydrogels prepared by TGase-catalyzed crosslinking reaction.

Based on the characteristics of the transglutaminase, various macromolecules with glutamine and lysine have been fabricated into polymer hydrogels via transglutaminase catalyzed reaction. Here, the main polymer hydrogels prepared by TGase catalyzed crosslinking are discussed in detail, as shown in Figure 2.

### Fibrin Hydrogels

Fibrin is a biopolymer that the end product of the physiological blood coagulation cascade, which plays a pivotal role in wound healing (Heher et al., 2018). Under physiological conditions, thrombin activates the transglutaminase Factor XIII (FXIIIa) and can further stabilizes the clot by cross-linking of fibrin polymers (Pieters and Wolberg, 2019). Therefore, fibrin hydrogels can be formed via the combination of fibrinogen and thrombin mixtures (Nelson and Gilbert, 2021). Abrego et al. (2022) compared the elastic properties of fibrin hydrogels and polyethylene glycol (PEG) hydrogels, and found an increase in the elasticity of the hydrogel with higher concentrations of fibrin and PEG, respectively. However, *in vitro* showed fast biodegradability and moderate mechanical properties, which limited the application in tissue engineering. Development of fibrin based composite hydrogels supplemented with different types polymer materials could be an effective strategy. Recently, Wang K. et al. (2021) combined cellulose nanocrystals (CNC) with fibrinogen and obtained CNC reinforced fibrin nanocomposite hydrogel, and the obtained hydrogel with improved mechanical stability showed an improvement in the formation of long myotubes (up to 800 μm).

### Polyethylene Glycol Hydrogels

PEG is a water-soluble and nontoxic synthetic polymer that has been broadly explored for biomedical applications. The coagulation factor XIII can prepare PEG hydrogel under physiological conditions without the addition of chemical initiators, one PEG precursor with a glutamine-containing sequence and the other one with a lysine-containing sequence can be coupled by factor XIII (Anjum et al., 2016). To overcome limitations of cell adhesion in the PEG hydrogel, cell-adhesion peptides (RGD) was utilized to conjugate onto the PEG polymer. The modified PEG hydrogels can alter adult dermal progenitor cells behavior and phenotype (Sparks et al., 2019). To advance the translation of human tissue analogues to the clinics, a hybrid hydrogel composed of gelatin and PEG were fabricated via factor XIII, and the hybrid hydrogel can be tailored by tuning the ratio between gelatin and PEG, the total polymer concentration and so on (Klotz et al., 2019).

### Soy Protein Hydrogels

As a kind of plant protein, soybean protein contains a complete variety of amino acids, especially lysine, essential for the human body. Hydrogels prepared from soybean protein have a broad application prospect in biomedicine, food and other fields. Tian and Liang (2005) used MTGase to promote soy protein isolate solution to form soy protein isolate gel and analyzed the effect of MTGase on soy protein isolate gel by changing enzyme dosage, pH value, reaction temperature, substrate protein concentration and reaction time. Song and Zhang (2008) hydrolyzed soybean protein isolate using MTGase and obtained protein-based hydrogels with adjustable gel time and mechanical strength. The hydrogel has further been applied for the controlled release of 5-amino salicylic acid. Guo et al. (2012) introduced glucan sulfate into soybean globulin to form a high-charge complex with glycine in protein and prepared transparent hydrogel with a stable network structure by MTGase. Comparably, layered montmorillonite nanoclay was intercalated into soy protein to enhance elastic properties of the hydrogel via TGase crosslinking (Jin and Zhong, 2013).

### Collagen Hydrogels

Collagen has excellent biocompatibility and biodegradability, and it is the main component of the extracellular matrix (ECM) of interstitial tissues (including skin, bone, cartilage, tendon and ligament). Orban et al. (2004) selectively mediated the chemical reaction between glutamine and lysine residues on protein fibers by MTGase, resulting in bovine collagen type I gel formation by the crosslinking reaction. Temperature-responsive and enzyme-responsive collagen hydrogels by MTGase were synthesized, and the enzyme concentrations could tune the degradation time and biocompatibility of the hydrogels (Zhao et al., 2016). Using 50 U/g MTGase, the prepared hydrogels had a high cross-linking degree and good resistance to collagenase I and collagenase II degradation. Lee et al. (2013) prepared collagen hydrogel with increased stiffness to promote more angiogenic sprouts that invade deeper, and the stiffness independent of ECM collagen concentration can be modulated by MTGase. Humanoid collagen hydrogels and fishbone collagen hydrogels based on MTGase and 1-ethyl-3-methylimidazolium acetate crosslinking were prepared, and the hydrogels could promote the proliferation of fibroblasts and inhibit the proliferation of cancer cells (Li and Fan, 2019).

### Gelatin Hydrogels

Gelatin is a protein derived from the hydrolysis of collagen with excellent biocompatibility and biodegradability. MTGase and gelatin can be dissolved in PBS (Phosphate Buffered Saline) solution and prepared gelatin hydrogel by the cross-linked reaction (Ren et al., 2016). Two types of gelatin (type-A and type-B) crosslinked by MTGase with different concentration were compared in physical and mechanical properties, and the type-A gelatin showed the superiority in crosslinking efficiency than type-B (Liu et al., 2020). Gelatin hydrogel encapsulated human adipocyte globules can increase the cell proliferation significantly with low cytotoxicity (Tsai et al., 2020). A sustained-release absorbable hydrogel by combining TGase with gelatin, alginate and antibiotics has developed for local delivery of antibiotics in orthopedic surgery (Sun et al., 2021). Gupta et al. (2021) used gelatin hydrogel prepared by MTGase crosslinking as an engineered skeletal muscle matrix, and the hydrogel was stable after 2 weeks under similar culture conditions of natural skeletal muscle. Tannic acid modified gelatin also can be crosslinked by TGase to form hydrogel, and the hydrogel exhibited comparable elasticity and flexibility, and therapeutic healing effects in the mouse skin incision and wound model *in vivo* (Zhou et al., 2021).

### Hyaluronic Acid Hydrogels

Hyaluronic acid (HA) is a natural polysaccharide in the extracellular matrix of numerous soft connective tissues, and best known for its intrinsic hydrating properties (Zamboni et al., 2021). Broguiere et al. (2016) introduced a hyaluronan hydrogel encapsulated neurons based on high molecular weight hyaluronic acid using activated transglutaminase factor XIII. At the same time, hyaluronic acid moieties was covalently linked to poly (ethylene glycol) macromer by activated transglutaminase factor XIII, leading to the formation of hybrid hydrogels (Ranga et al., 2016). In comparison to pure PEG hydrogel or HA hydrogel, the hybrid hydrogel can afford ideal attributes of both materials with minimizing macrophage infiltration *in vivo*, which was suited for bone marrow organoid formation (Vallmajo-Martin et al., 2020). Similarly, hyaluronic acid-fibrin hydrogel loaded with lipophilic anti-inflammatory drugs has been formed *in situ* by factor XIII, and the hydrogel can be a drug delivery system for intra-articluar administration (Storozhylova et al., 2020).

## Concluding Remarks

In this work, TGase catalyzed reactions for the synthesis of polymer hydrogels were reviewed. As outlined, various polymer materials including fibrin, polyethylene glycol, soybean protein, collagen, gelatin and hyaluronic acid have been successfully catalyzed to form hydrogels by the TGase. More importantly, the TGase can offer tight integration between the formed hydrogel and the native tissue, making it feasible to improve their biocompatibility of the hydrogel. Despite the significant advances of using the TGase catalyzed strategy, there is still much room for improvement. For example, the mechanical properties of some hydrogels by TGase are poor or limited for medical application. In particular, large-scale production of hydrogel with high intensity and strength is still a challenge. For the diverse biomedical fields, the polymer hydrogel with functional groups and higher biocompatibility prepared by TGase is deficient. In practical clinical application, the safety and functionality of the hydrogel materials is extremely crucial. It is believed that a massive effort in this exciting field will be made, and continuous breakthroughs will be made with further research. The enzyme catalysis provides a powerful pool to synthesize polymer hydrogel with selective bioprocess, and the enzyme-catalyzed hydrogels are expected to be the next generation of biomaterials for tissue engineering and regenerative medicine.

## References

[B1] Abrego C. J. G. Dedroog L. Deschaume O. Wellens J. Vananroye A. Lettinga M. P. (2022). Multiscale Characterization of the Mechanical Properties of Fibrin and Polyethylene Glycol (PEG) Hydrogels for Tissue Engineering Applications. Macro Chem. Phys. 223 (1), 2100366. 10.1002/macp.202100366

[B2] Anjum F. Lienemann P. S. Metzger S. Biernaskie J. Kallos M. S. Ehrbar M. (2016). Enzyme Responsive GAG-Based Natural-Synthetic Hybrid Hydrogel for Tunable Growth Factor Delivery and Stem Cell Differentiation. Biomaterials 87, 104–117. 10.1016/j.biomaterials.2016.01.050 26914701

[B3] Behabtu N. Kralj S. (2020). Enzymatic Polymerization Routes to Synthetic-Natural Materials: A Review. ACS Sustain. Chem. Eng. 8, 9947–9954. 10.1021/acssuschemeng.0c01664

[B4] Broguiere N. Isenmann L. Zenobi-Wong M. (2016). Novel Enzymatically Cross-Linked Hyaluronan Hydrogels Support the Formation of 3D Neuronal Networks. Biomaterials 99, 47–55. 10.1016/j.biomaterials.2016.04.036 27209262

[B5] Cui R. Wu Q. Wang J. Zheng X. Ou R. Xu Y. (2021). Hydrogel-By-Design: Smart Delivery System for Cancer Immunotherapy. Front. Bioeng. Biotechnol. 9, 723490. 10.3389/fbioe.2021.723490 34368109 PMC8334721

[B6] Duarte L. Matte C. R. Bizarro C. V. Ayub M. A. Z. (2020). Review Transglutaminases: Part II-Industrial Applications in Food, Biotechnology, Textiles and Leather Products. World J. Microbiol. Biotechnol. 36, 11. 10.1007/s11274-019-2792-9 31879822

[B7] Fatima S. W. Khare S. K. (2018). Current Insight and Futuristic Vistas of Microbial Transglutaminase in Nutraceutical Industry. Microbiol. Res. 215, 7–14. 10.1016/j.micres.2018.06.001 30172311

[B8] Fisher S. A. Baker A. E. G. Shoichet M. S. (2017). Designing Peptide and Protein Modified Hydrogels: Selecting the Optimal Conjugation Strategy. J. Am. Chem. Soc. 139 (22), 7416–7427. 10.1021/jacs.7b00513 28481537

[B9] Gharibzahedi S. M. T. Roohinejad S. George S. Barba F. J. Greiner R. Barbosa-Cánovas G. V. (2018). Innovative Food Processing Technologies on the Transglutaminase Functionality in Protein-Based Food Products: Trends, Opportunities and Drawbacks. Trends Food Sci. Techn. 75, 194–205. 10.1016/j.tifs.2018.03.014

[B10] Guebitz G. M. Nyanhongo G. S. (2018). Enzymes as green Catalysts and Interactive Biomolecules in Wound Dressing Hydrogels. Trends Biotechnol. 36, 1040–1053. 10.1016/j.tibtech.2018.05.006 29914650

[B11] Guo J. Zhang Y. Yang X.-Q. (2012). A Novel Enzyme Cross-Linked Gelation Method for Preparing Food Globular Protein-Based Transparent Hydrogel. Food Hydrocolloids 26 (1), 277–285. 10.1016/j.foodhyd.2011.06.005

[B12] Gupta D. Santoso J. W. McCain M. L. (2021). Characterization of Gelatin Hydrogels Cross-Linked with Microbial Transglutaminase as Engineered Skeletal Muscle Substrates. Bioengineering 8 (1), 6. 10.3390/bioengineering8010006 33418892 PMC7825108

[B13] Heher P. Mühleder S. Mittermayr R. Redl H. Slezak P. (2018). Fibrin-based Delivery Strategies for Acute and Chronic Wound Healing. Adv. Drug Deliv. Rev. 129, 134–147. 10.1016/j.addr.2017.12.007 29247766

[B14] Jin M. Zhong Q. (2013). Transglutaminase Cross-Linking to Enhance Elastic Properties of Soy Protein Hydrogels with Intercalated Montmorillonite Nanoclay. J. Food Eng. 115, 33–40. 10.1016/j.jfoodeng.2012.09.016

[B15] Klotz B. J. Oosterhoff L. A. Utomo L. Lim K. S. Vallmajo‐Martin Q. Clevers H. (2019). A Versatile Biosynthetic Hydrogel Platform for Engineering of Tissue Analogues. Adv. Healthc. Mater. 8 (19), 1900979. 10.1002/adhm.201900979 PMC7116179 31402634

[B16] Lai E. Yue X. Ning W. e. Huang J. Ling X. Lin H. (2019). Three-dimensional Graphene-Based Composite Hydrogel Materials for Flexible Supercapacitor Electrodes. Front. Chem. 7, 660. 10.3389/fchem.2019.00660 31632952 PMC6779856

[B17] Lee P.-F. Bai Y. Smith R. L. Bayless K. J. Yeh A. T. (2013). Angiogenic Responses Are Enhanced in Mechanically and Microscopically Characterized, Microbial Transglutaminase Crosslinked Collagen Matrices with Increased Stiffness. Acta Biomater. 9 (7), 7178–7190. 10.1016/j.actbio.2013.04.001 23571003 PMC3749884

[B18] Li P. Zhong Y. Wang X. Hao J. (2020). Enzyme-regulated Healable Polymeric Hydrogels. ACS Cent. Sci. 6, 1507–1522. 10.1021/acscentsci.0c00768 32999926 PMC7517121

[B19] Li X. Fan D. (2019). Smart Collagen Hydrogels Based on 1-Ethyl-3-Methylimidazolium Acetate and Microbial Transglutaminase for Potential Applications in Tissue Engineering and Cancer Therapy. ACS Biomater. Sci. Eng. 5 (7), 3523–3536. 10.1021/acsbiomaterials.9b00393 33405735

[B20] Liu Y. Weng R. Wang W. Wei X. Li J. Chen X. (2020). Tunable Physical and Mechanical Properties of Gelatin Hydrogel after Transglutaminase Crosslinking on Two Gelatin Types. Int. J. Biol. Macromolecules 162, 405–413. 10.1016/j.ijbiomac.2020.06.185 32574738

[B21] Maddock R. M. A. Pollard G. J. Moreau N. G. Perry J. J. Race P. R. (2020). Enzyme-catalysed Polymer Cross-Linking: Biocatalytic Tools for Chemical Biology, Materials Science and beyond. Biopolymers 111, e23390. 10.1002/bip.23390 32640085

[B22] Miwa N. (2020). Innovation in the Food Industry Using Microbial Transglutaminase: Keys to success and Future Prospects. Anal. Biochem. 597, 113638. 10.1016/j.ab.2020.113638 32097607

[B23] Nelson D. W. Gilbert R. J. (2021). Extracellular Matrix‐Mimetic Hydrogels for Treating Neural Tissue Injury: A Focus on Fibrin, Hyaluronic Acid, and Elastin‐Like Polypeptide Hydrogels. Adv. Healthc. Mater. 10 (22), 2101329. 10.1002/adhm.202101329 PMC8599642 34494398

[B24] Orban J. M. Wilson L. B. Kofroth J. A. El-Kurdi M. S. Maul T. M. Vorp D. A. (2004). Crosslinking of Collagen Gels by Transglutaminase. J. Biomed. Mater. Res. 68A (4), 756–762. 10.1002/jbm.a.20110 14986330

[B25] Pieters M. Wolberg A. S. (2019). Fibrinogen and Fibrin: an Illustrated Review. Res. Pract. Thromb. Haemost. 3, 161–172. 10.1002/rth2.12191 31011700 PMC6462751

[B26] Qin X. S. Jiang S. T. Zhao Y. Y. Zheng Z. (2017). Effect of Wheat Gluten on Gelation Properties of Soy Protein Isolate Induced by Transglutaminase Crosslinking. Sci. Technol. Food Ind. 38 (8), 214–217+243. 10.13386/j.issn1002-0306.2017.08.033

[B27] Ranga A. Lutolf M. P. Hilborn J. Ossipov D. A. (2016). Hyaluronic Acid Hydrogels Formed *In Situ* by Transglutaminase-Catalyzed Reaction. Biomacromolecules 17 (5), 1553–1560. 10.1021/acs.biomac.5b01587 27014785

[B28] Ren X. M. Qian H. Xiao Z. H. Long H. Y. Guo Y. Q. Yang G. (2016). Three-dimensional Cultured Adipose-Derived Stem Cells Based on Microbial Transglutaminase Enzyme Crosslinked Gelatin Hydrogel. Chin. J. Rep. Reconst. Sur. 30 (12), 1532–1537. 10.7507/1002-1892.20160316 29786347

[B29] Savoca M. Tonoli E. Atobatele A. Verderio E. (2018). Biocatalysis by Transglutaminases: a Review of Biotechnological Applications. Micromachines 9 (11), 562. 10.3390/mi9110562 PMC6265872 30715061

[B30] Schmitz T. Bäuml C. A. Imhof D. (2020). Inhibitors of Blood Coagulation Factor XIII. Anal. Biochem. 605, 113708. 10.1016/j.ab.2020.113708 32335064

[B31] Sharma S. Tiwari S. (2020). A Review on Biomacromolecular Hydrogel Classification and its Applications. Int. J. Biol. Macromolecules 162, 737–747. 10.1016/j.ijbiomac.2020.06.110 32553961

[B32] Song F. Zhang L.-M. (2008). Enzyme-catalyzed Formation and Structure Characteristics of a Protein-Based Hydrogel. J. Phys. Chem. B 112 (44), 13749–13755. 10.1021/jp8041389 18855437

[B33] Sparks H. D. Anjum F. Vallmajo-Martin Q. Ehrbar M. Abbasi S. Kallos M. S. (2019). Flowable Polyethylene Glycol Hydrogels Support the *In Vitro* Survival and Proliferation of Dermal Progenitor Cells in a Mechanically Dependent Manner. ACS Biomater. Sci. Eng. 5 (2), 950–958. 10.1021/acsbiomaterials.8b01294 33405787

[B34] Storozhylova N. Crecente-Campo J. Cabaleiro D. Lugo L. Dussouy C. Simões S. (2020). An *In Situ* Hyaluronic Acid-Fibrin Hydrogel Containing Drug-Loaded Nanocapsules for Intra-articular Treatment of Inflammatory Joint Diseases. Regen. Eng. Transl. Med. 6, 201–216. 10.1007/s40883-020-00154-2

[B35] Sun C.-K. Ke C.-J. Lin Y.-W. Lin F.-H. Tsai T.-H. Sun J.-S. (2021). Transglutaminase Cross-Linked Gelatin-Alginate-Antibacterial Hydrogel as the Drug Delivery-Coatings for Implant-Related Infections. Polymers 13 (3), 414. 10.3390/polym13030414 33525449 PMC7866112

[B36] Tian S. J. Liang H. M. (2005). Effect of Glutaminase on Gelatabiligy of Soybean Protein Isolatezhon. Chin. Oil. Fat. 30 (8), 43–46. 10.3321/j.issn:1003-7969.2005.08.014

[B37] Tsai C.-C. Kuo S.-H. Lu T.-Y. Cheng N.-C. Shie M.-Y. Yu J. (2020). Enzyme-Cross-linked Gelatin Hydrogel Enriched with an Articular Cartilage Extracellular Matrix and Human Adipose-Derived Stem Cells for Hyaline Cartilage Regeneration of Rabbits. ACS Biomater. Sci. Eng. 6 (9), 5110–5119. 10.1021/acsbiomaterials.9b01756 33455262

[B38] Vallmajo-Martin Q. Broguiere N. Millan C. Zenobi-Wong M. Ehrbar M. (2020). PEG/HA Hybrid Hydrogels for Biologically and Mechanically Tailorable Bone Marrow Organoids. Adv. Funct. Mater. 30 (48), 1910282. 10.1002/adfm.201910282

[B39] Varma I. K. Albertsson A.-C. Rajkhowa R. Srivastava R. K. (2005). Enzyme Catalyzed Synthesis of Polyesters. Prog. Polym. Sci. 30, 949–981. 10.1016/j.progpolymsci.2005.06.010

[B40] Wang J. Ding Y. Jiang Y. Dong H. Wang Q. Cheng J. (2021). Progress in Understanding the Effect of Enzymatic Modification on Gel Properties of Soy Protein Isolate. Food Sci. 15, 329–336. 10.7506/spkx1002-6630-20200602-023

[B41] Wang K. Mosser G. Haye B. Baccile N. Le Griel P. Pernot P. (2021). Cellulose Nanocrystal-Fibrin Nanocomposite Hydrogels Promoting Myotube Formation. Biomacromolecules 22 (6), 2740–2753. 10.1021/acs.biomac.1c00422 34027656

[B42] Xue T. Zheng X. Su X. Chen D. Liu K. Yuan X. (2020). Directed Evolution of the Transglutaminase from S*treptomyces Mobaraensis* and its Enhanced Expression in *Escherichia coli* . Food Biotechnol. 34 (1), 42–61. 10.1080/08905436.2019.1711112

[B43] Yan J. Bockstaller M. R. Matyjaszewski K. (2020). Brush-modified Materials: Control of Molecular Architecture, Assembly Behavior, Properties and Applications. Prog. Polym. Sci. 100, 101180. 10.1016/j.progpolymsci.2019.101180

[B44] Zamboni F. Okoroafor C. Ryan M. P. Pembroke J. T. Strozyk M. Culebras M. (2021). On the Bacteriostatic Activity of Hyaluronic Acid Composite Films. Carbohydr. Polym. 260, 117803. 10.1016/j.carbpol.2021.117803 33712151

[B45] Zhao L. Li X. Zhao J. Ma S. Ma X. Fan D. (2016). A Novel Smart Injectable Hydrogel Prepared by Microbial Transglutaminase and Human-like Collagen: Its Characterization and Biocompatibility. Mater. Sci. Eng. C 68, 317–326. 10.1016/j.msec.2016.05.108 27524026

[B46] Zhou J. Wu Y. Zhang X. Lai J. Li Y. Xing J. (2021). Enzyme Catalyzed Hydrogel as Versatile Bioadhesive for Tissue Wound Hemostasis, Bonding, and Continuous Repair. Biomacromolecules 22 (4), 1346–1356. 10.1021/acs.biomac.0c01329 33657790

